# Establishment of a High-Throughput Assay to Monitor Influenza A Virus RNA Transcription and Replication

**DOI:** 10.1371/journal.pone.0133558

**Published:** 2015-07-21

**Authors:** Zhen Wang, Fei Zhao, Qian Gao, Zhenlong Liu, Yongxin Zhang, Xiaoyu Li, Yuhuan Li, Weilie Ma, Tao Deng, Zhizhen Zhang, Shan Cen

**Affiliations:** 1 Institute of Medicinal Biotechnology, Chinese Academy of Medical Sciences & Pekin Union Medical College, Beijing, China; 2 Department of Biochemistry, Guangdong Medical College, Zhanjiang, Guangdong, China; 3 Institute of Pathogen Biology, Chinese Academy of Medical Sciences & Pekin Union Medical College, Beijing, China; University of Edinburgh, UNITED KINGDOM

## Abstract

Influenza A virus (IAV) poses significant threats to public health because of the recent emergence of highly pathogenic strains and wide-spread resistance to available anti-influenza drugs. Therefore, new antiviral targets and new drugs to fight influenza virus infections are needed. Although IAV RNA transcription/replication represents a promising target for antiviral drug development, no assay ideal for high-throughput screening (HTS) application is currently available to identify inhibitors targeting these processes. In this work, we developed a novel HTS assay to analyze the transcription and replication of IAV RNA using an A549 cell line stably expressing IAV RNA-dependent RNA polymerase (RdRp) complex, NP and a viral mini-genomic RNA. Both secreted *Gaussia* luciferase (Gluc) and blasticidin resistance gene (Bsd) were encoded in the viral minigenome and expressed under the control of IAV RdRp. Gluc serves as a reporter to monitor the activity of IAV RdRp, and Bsd is used to maintain the expression of all foreign genes. Biochemical studies and the statistical analysis presented herein demonstrate the high specificity, sensitivity and reproducibility of the assay. This work provides an ideal HTS assay for the identification of inhibitors targeting the function of IAV RdRp and a convenient reporting system for mechanism study of IAV RNA transcription / replication.

## Introduction

Influenza A virus (IAV) causes contagious respiratory disease resulting in hospitalization and even death. The unprecedented emergence of highly pathogenic avian influenza A (H5N1) in 2005 and worldwide outbreak of the swine-originated influenza virus A (H1N1) in 2009 aroused serious concerns on the health threat posed by genetic variation of this virus. Although vaccination remains the primary method to protect people from viral infection, antigenic drift and shift in IAV limit the effectiveness of vaccination [1]. Currently, in most countries only two classes of anti-influenza drugs are available for clinical therapy, M2 ion channel blockers and neuraminidase inhibitors [2]. However, high percentages of circulating IAV strains have developed resistance to these drugs via frequently mutation of M2 and neuraminidase targets [3–5]. Thus, new anti-influenza targets and drugs are urgently needed.

Influenza viruses are the family members of *orthomyxoviridae* and include A, B and C types. Among three types of influenza viruses, IAV is responsible for the outbreaks of all pandemic influenza, which contains 8 segmented, negative-sense and single-stranded genome [6]. Each vRNA segment is bound to viral NP proteins and a copy of RNA-dependent RNA polymerase (RdRp) to form viral ribonucleoprotein (vRNP) complexes. RdRp is a heterotrimeric complex consisting of viral PB2, PB1 and PA subunits and catalyzes the synthesis of viral mRNA and vRNA via an intermediate complementary RNA (cRNA). In infected cells, vRNPs are transported to the nucleus and initiate viral genomic transcription and replication. In the nucleus, the 5’ and 3’ ends of vRNA binds to influenza RdRp complex and activates the synthesis of viral mRNA and cRNA. Then cRNA is used as a template to synthesize vRNA (reviewed in [7]). In addition to the above viral proteins, multiple host factors are also involved in these processes [8–12]. Clearly, IAV genomic transcription and replication are pivotal in viral life cycle and RdRp plays a central role in these processes. Moreover, IAV RdRp exhibits relatively conserved among all IAV proteins and different mode of action from human RNA polymerases. All these facts together make it a promising anti-influenza drug target.

Although several attempts have been done to search for inhibitors targeting IAV RNA transcription/replication [13–17], the development of anti-IAV drug in this category is still hindered by the lack of an efficient assay suitable for high-throughput screening (HTS). Using plasmid transfection, transient expression of influenza RdRp complex, NP and vRNA is able to reconstitute an IAV minigenome transcription/replication system in cell [13, 16–24]. However, the feasibility and variability of the transient assay greatly limits its use in large-scale compound screening. An early work showed one cell line (3PNP-4) stably expressing the three viral polymerase proteins and the NP [25]. A recent work reported a 293 cell line stably expressing influenza vRNPs, in which a drug resistance gene on the virus-like RNA was used to monitor the activity of IAV RdRp [26]. Due to the low detection sensitivity of drug resistance selection, there is a clear need for a better strategy.

In our study, we developed a novel HTS assay for screening inhibitors targeting IAV RNA transcription/replication using an A549 cell line stably expressing IAV RdRp complex, NP and a viral mini-genomic RNA. In the assay, *Gaussia* luciferase (Gluc), a secreted luciferase and blasticidin resistance gene (Bsd), both of which were encoded in the viral minigenome, were expressed dependent on IAV RdRp. Sensitive Gluc was served as a reporter to monitor the activity of IAV RdRp, and Bsd was used to maintain the expression of all of the foreign genes. The validation analysis presented herein demonstrated that this assay could be used for HTS of novel anti-IAV drugs and mechanism study on IAV RNA transcription/replication.

## Materials and Methods

### Cells

A549 cells (ATCC) were cultured in Dulbecco’s modified Eagle’s medium (DMEM; Gibco) containing 10% fetal bovine serum (FBS; Gibco). 293FT cells (Life Technologies) were cultured in DMEM supplemented with 2 mM L-glutamix (Gibco), 0.1 mM MEM non-essential amino acids (Gibco) and 10% FBS. All cells were maintained at 37°C in 5% CO_2_.

### Plasmids construction

Lentiviral expression vector pWPXLd (Addgene) was inserted sequences encoding internal ribosome entry site (IRES) and puromycin-resistance gene into SpeI/NdeI sites to generate pWPXLd-puro vector. To obtain pWPXLd-puro based plasmids expressing PB2, PB1, PA and NP proteins, the opening reading frames of each protein from influenza strain A/WSN/33 (H1N1) was cloned into pWPXLd-puro. To construct plasmid pWPXLd-Gluc-IRES-Bsd, which produced negative strand of vRNA encoding *Gaussia* luciferase (Gluc) and blasticidin-resistance gene (Bsd), IRES/Bsd fragment was firstly constructed by PCR amplification of sequences and ligated by overlap PCR and then inserted into XbaI site of pHH21-Gluc (kindly provided by Dr. Erik de Vries [27]) to generate pHH21-Gluc-IRES-Bsd. The whole transcriptional region in pHH21-Gluc-IRES-Bsd, from human RNA polymerase I (pol I) promoter to the mouse pol I terminator including Gluc/IRES/Bsd expression cassette in anti-sense flanked by 5’ and 3’ untranslated regions of IAV NP segment, was cloned into BamHI/EcoRI sites of pWPXLd-puro. All plasmid constructs were sequenced to avert unwanted mutations.

### Lentivirus production and transduction

To produce lentiviral particles carrying PB2, PB1, PA, NP and Gluc/Bsd gene, pWPXLd-based plasmids together with the packaging plasmids pMD2.G (Addgene) and psPAX2 (Addgene) were co-transfected into 293FT cells using Lipofectamine 2000 transfection reagent (Life Technologies). 48 hours later, lentivirus-containing supernatants were harvested and filtered through 0.45um filter. Lentiviruses subsequently infected A549 cells in the presence of 8ug/mL polybrene. At 8 hours post infection, supernatants were replaced with fresh medium, followed by selection with blasticidin. By limiting dilution, a single cell clone exhibiting a high level of Gluc expression was obtained and named A549-5Ps. For A549-GLuc, A549 cells were conducted with lentivirus-containing Gluc/Bsd gene, and resultant cell line only contains the anti-sense Gluc/IRES/Bsd expression cassette without other components of RdRp.

shRNAs used in our experiments, were ordered from Sigma Aldrich. sheGPF (Catalog No: SHC005) is specific for GFP and was used as a negative control. To produce lentiviral particles expressing shRNA, shRNA based plasmids together with the packaging plasmids pMD2.G and psPAX2 were co-transfected into 293FT cells using Lipofectamine 2000 transfection reagent (Life Technologies).

### RT-PCR analysis of viral mRNAs

Total RNAs from A549 and A549-5Ps cells were extracted by TRIzol reagent (Life Technologies). RNAs were converted to cDNA by M-MLV Reverse Transcriptase (Promega) using random primers (Takara) and amplified by PCR with specific primers. The primer pairs used for PCR amplification were as follows: PB2 forward 5’-ACT GGT CCG CAA AAC GAG ATT C-3’, reverse 5’-CAT TGA CAT CTC GGT GCT TGG-3’; PB1 forward 5’-ATG GAT GTC AAT CCG ACT TT-3’, reverse 5’-CTA TTT TTG CCG TCT GAG CTC-3’; NP forward 5’-ATG GCG ACC AAA GGC ACC AAA C-3’, reverse 5’-TTA ATT GTC GTA CTC TG-3’; model vRNA forward 5’-CAC TCA CTG AGT GAC ATC GG-3’, reverse 5’-TTA GCC CTC CCA CAC ATA AC-3’. PCR products were purified by gel extraction and sequenced to confirm the amplified sequences.

### Small interfering RNA (siRNA)-mediated knockdown

A549-5Ps cells (5×10^4^/well of a 24-well plate) were transfected with siRNA using Lipofectamine RNAiMAX transfection reagent (Life Technologies) by forward transfection according to manufacturer’s instructions. Supernatants were replaced with fresh medium at 72 hours post transfection and cells were cultured for an additional 24 hours. Supernatants were collected for Gluc activity measurement and cells were harvested for the extraction of total cellular RNAs. RNA quantification was carried out using One Step SYBR PrimeScript RT-PCR Kit II (Takara) in Mx3000P system (Stratagene). The amplification process started with reverse transcription step at 42°C for 5min and 95°C for 10s, followed by 40 cycles for PCR amplification at 95°C for 5s, 60°C for 34s, followed by a melting curve analysis to confirm the specificity of amplification. The siRNA sequences used in the experiment were as follows: PB1 sense, 5’- CAA UGA UCU UGG UCC AGCA -3’; PB2 sense, 5’-cAA gca gUg UgU aca UUgA-3’; M2 sense, 5’-ACA GCA GAA UGC UGU GGAU-3’; cyclinT1 sense 5'-UCC CUU CCU GAU ACU AGA AdTdT-3'; Tat-SF1 sense, 5'-CAG GCC AAU UAU GGC UUC UTT-3'. A commercial scramble siRNA (Ruibo Inc.) was used as a control. Real-time RT-PCR primers used in the experiment were as follows: PB1 forward 5’-CTG CCA GAA GAC AAT GAA CC-3’, reverse 5’-GGC CAT TGC TTC CAA TAC AC-3’; PB2 forward 5’-GCA GAA CCC AAC AGA AGA GC-3’, reverse 5’-GCT GTT GCT CTT CTC CCA AC-3’; cyclinT1 forward 5’-TTG TTC GAG CAA GCA AGG ACT-3’, reverse 5’-CCG TCA GTT GAG ACT GGG AT-3’; Tat-SF1 forward 5’-TCT GTG GAA CTT GCA TTA AAA CTT TTG-3’, reverse 5’-CAG CTT CTT CTT ATA GTC TTT GCA C-3’; GAPDH forward 5’- GGT ATC GTG GAA GGA CTC ATG AC-3’, reverse 5’- ATG CCA GTG AGC TTC CCG TTC AG-3’. To further validate the effect of PB2 siRNA, the expression of PB2 was analyzed by Western Blot using an antibody specific for PB2, which was kindly provided by Tao Deng. As a loading control, β-actin was analyzed using an anti- β-actin antibody (Abcam).

### Cell viability assay

Cell viability was assessed by Cell Counting Kit-8 (CCK-8; Beyotime) using a water soluble tetrazolium salt-8 (WST-8). Cell supernatants were replaced with fresh medium containing CCK-8 reagent (110uL medium containing 10uL CCK-8 reagent per well) and incubated for 1 hour at 37°C with 5% CO_2_. The absorbance at 450 nm was subsequently measured using EnSpire 2300 Multilable Reader (PerkinElmer).

### Gluc activity assay

Gluc activity was measured according to the method described by Tannous [28]. Firstly, a solution of 16.7uM coelenterazine-h dissolved in PBS was incubated in the dark for 30min at room temperature. Secondly, 40uL supernatant was transferred to a well of white and opaque 96-well plate. Finally, 60uL of 16.7uM coelenterazine-h was injected to acquire photon counts for 0.5s using Centro XS^3^ LB 960 microplate luminometer (BERTHOLD TECHNOLOGIES).

### Statistical analysis

Statistical significance analysis was assessed by the Tukey test and T test. The Z’ value was calculated as follows: Z’ = 1 − (3 × SD of positive control + 3 × SD of negative control)/(mean of positive control − mean of negative control), where SD represents the standard deviation. Z’ value between 0.5 and 1.0 is considered robust enough for an HTS assay.

## Results

### Establishment of lentiviral vector-based IAV minigenome transcription/replication system

**Fig 1:**
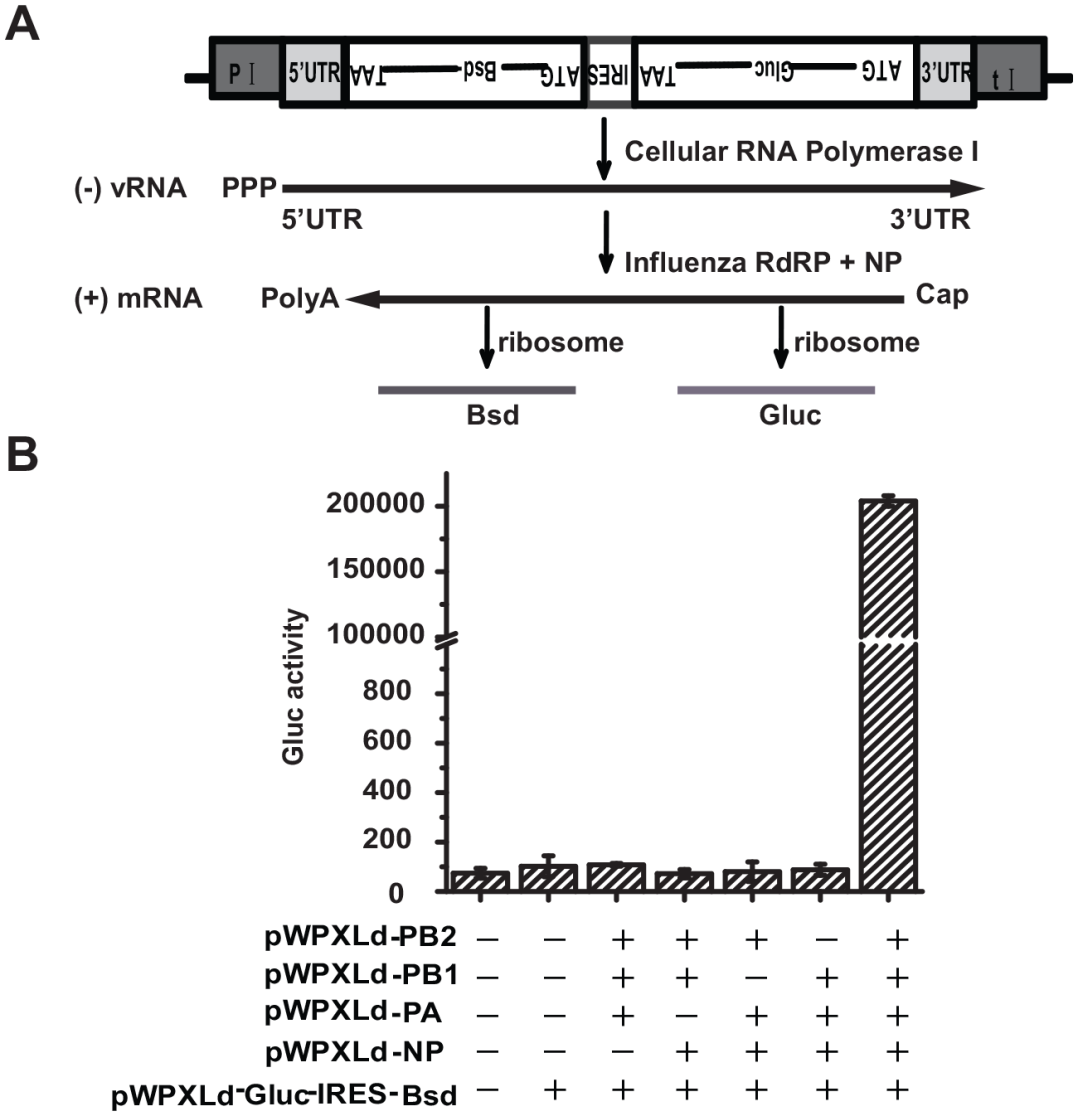
Construction of Gluc/Bsd reporter system driven by influenza RdRp. (A) Schematic representation of Gluc/Bsd reporter system. Gluc/Bsd reporter construct comprises Gluc open reading frame and Bsd open reading frame, separated by IRES element. The expression cassette in anti-sense, which flanked by 5’ and 3’ untranslated regions (UTRs) of IAV NP segment, is inserted into the human pol I promoter (P I) and the mouse pol I terminator (t I). Driven by cellular pol I, negative-sense model vRNA is synthesized. Subsequently, model vRNA binding NP molecules is transcripted into mRNA depended on influenza RdRp. Gluc reporter gene is then translated into Gluc protein and blasticidin resistance is achieved. (B) Gluc expression in Gluc/Bsd reporter system depended on influenza RdRp in the presence of NP protein. 293T cells (4×10^5^) were co-transfected with the indicated plasmid combinations (0.1μg each plasmid). At 48 hours post-transfection, supernatants were harvested and subjected to Gluc activity assay. Error bars standard error of the mean of three independent experiments.

To establish an IAV minigenome transcription/replication system, we first constructed a lentiviral vector expressing both Gluc and Bsd, in which these two genes were separated by an internal ribosome entry site (IRES) element (Fig 1A). In the construct, the anti-sense Gluc/IRES/Bsd expression cassette is flanked by 5’ and 3’ untranslated regions (UTRs) of the IAV NP segment and is inserted between the human RNA polymerase I (pol I) promoter and the mouse pol I terminator. Upon introduction of the lentiviral vector into cells, a negative-sense vRNA is synthesized by cellular pol I and subsequently serves as a template for mRNA transcription by IAV RdRp (Fig 1A). Such a design ensures that the expression cassette functions as an IAV minigenome, in which Gluc/Bsd can only be expressed as dependent on the activity of IAV RdRp in the presence of NP. Therefore, the content of Gluc secreted into a culture medium reflects the activity of RdRp, and Bsd can be used as a selective marker for constitutive expression of all components of the IAV minigenome transcription/replication system. To constitute this system, a series of lentiviral plasmids expressing PB2, PB1, PA and NP were also constructed, respectively.

To initially validate our strategy, 293T cells were co-transfected with different combinations of plasmids expressing Gluc/Bsd, PB2, PB1, PA and NP, and Gluc activity was measured at 48 hours post transfection. As anticipated, Gluc was only expressed in the cells co-transfected with all five plasmids (Fig 1B). This initially demonstrates that the expression of the Gluc reporter is specifically driven by influenza RdRp.

### Generation of A549-derived cell lines stably expressing IAV minigenome transcription/replication system

**Fig 2:**
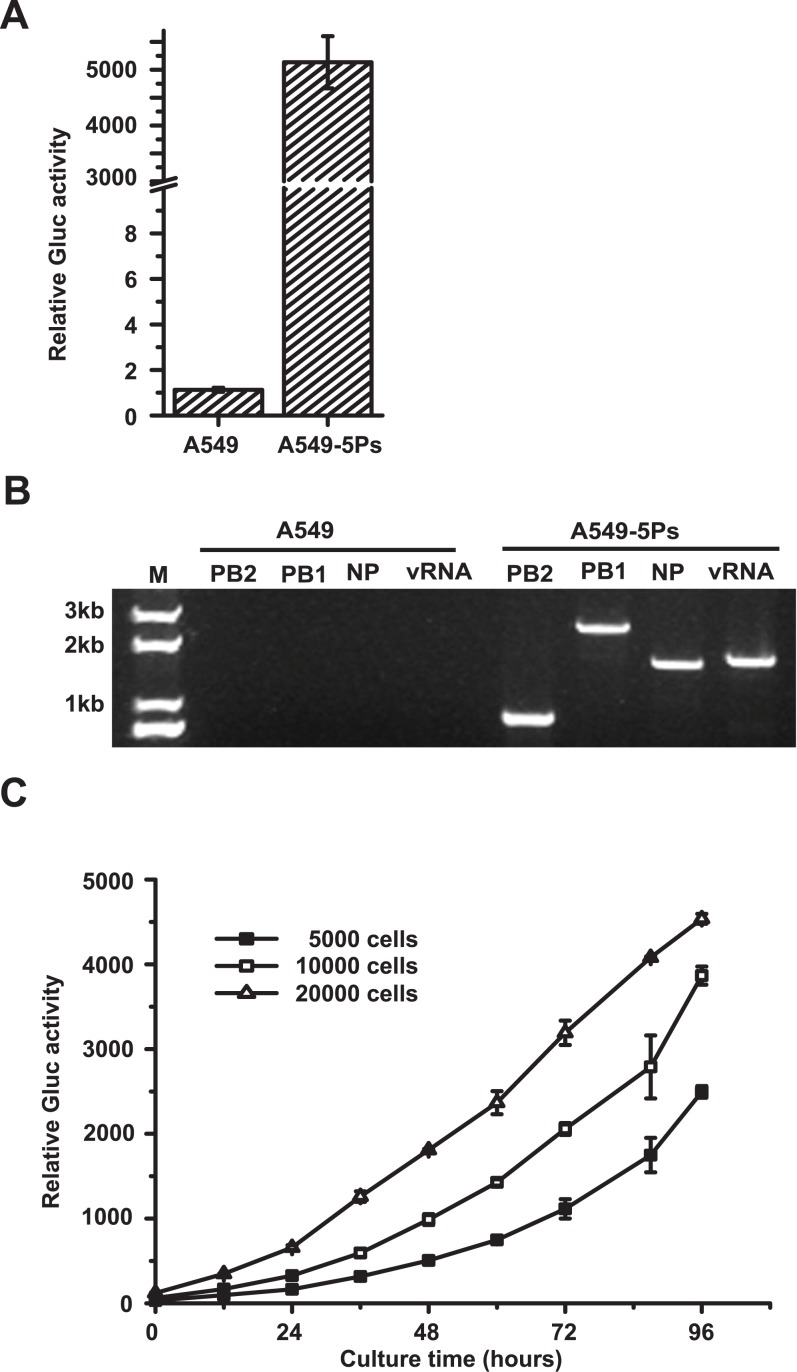
Characterization of A549-5Ps cells. (A-B) Stable expression of influenza A virus minigenome transcription/replication system in A549-5Ps cells. A549 and A549-5Ps cells were seeded in 6-well plates and cultured for 48 hours. (A) Gluc activity in supernatants was quantified. (B) Total cellular RNAs were extracted and then subjected to RT-PCR using primer pairs specific for PB2, PB1, NP and model vRNA. (C) Relative Gluc activity over a 96-hour time course at different A549-5Ps cell numbers. A549 and A549-5Ps cells were seeded in 96-well plates (5,000/well, 10,000/well, 20,000/well) and supernatants were harvested at different time points, followed by Gluc activity measurement. Error bars standard error of the mean of three independent experiments.

To establish a cell line constitutively expressing the IAV minigenome transcription/replication system, A549 cells were transducted with VSV-G pseudotyped lentiviral vectors that expressed the Gluc/Bsd reporter, NP, PA, PB1 and PB2, respectively, followed by selection with blasticidin. By limiting dilution, we obtained a single cell clone exhibiting a high level of Gluc expression (Fig 2A), which was named A549-5Ps. RT-PCR analysis of total cellular RNAs showed mRNA production of PB1, PB2 and NP, as well as the vRNA in A549-5Ps cells (Fig 2B), which were further validated by DNA sequencing. These data collectively demonstrate that the IAV minigenome coding for Gluc/Bsd and viral proteins required for vRNA transcription are stably expressed in A549-5Ps cells. Furthermore, parent A549 cells and A549-5Ps cells showed a similar proliferation rate, suggesting that the stable expression of the IAV minigenome and viral proteins has no significant effect on the proliferation and viability of A549-5Ps cells (results not shown).

To further characterize the A549-5Ps cells, we assessed the activity of Gluc over a 96-hour time course right after different numbers (5,000, 10,000 and 20,000 of cells per well) of the cells were seeded. Results showed that the activity of Gluc in supernatant increased over the time course (Fig 2C). Notably, over 24 hours the fluorescence intensity reached to a level of 165-658-fold above that of A549 cells. This demonstrates an impressive detection sensitivity of the assay, showing its potential in developing a convenient system to monitor the activity of IAV RdRp.

### Functional validation of the IAV minigenome transcription/replication system

**Fig 3:**
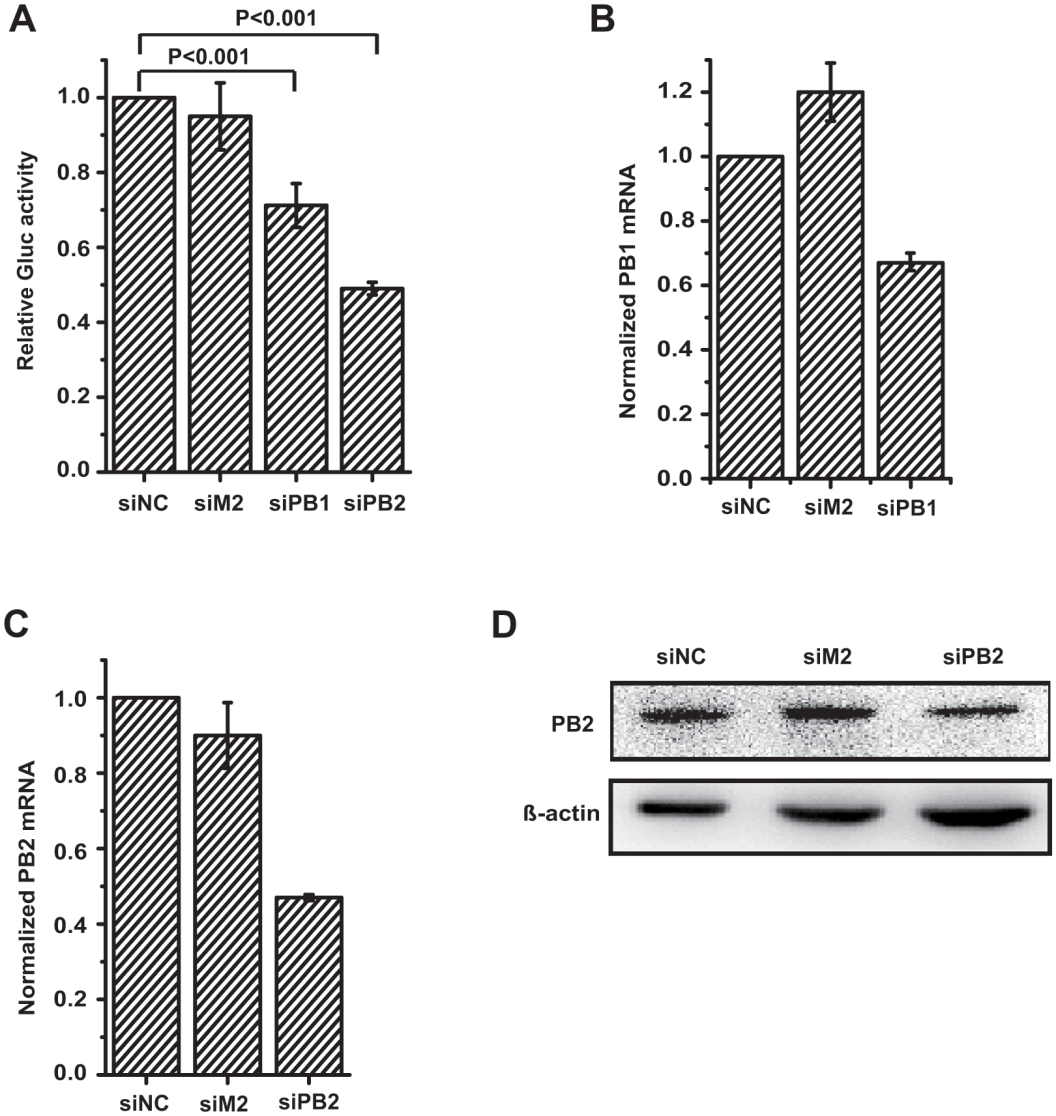
Effect of PB1 and PB2 knockdown on Gluc expression in A549-5Ps cells. A549-5Ps cells were transfected with 30nM negative control siRNA (NC), PB1 siRNA, PB2 siRNA and irrelevant control M2 siRNA, respectively, as described in materials and methods. At 72 hours post transfection, supernatants were replaced with fresh medium and cells were cultured for a further 24 hours. (A) Gluc activity in supernatants was quantified. Total cellular RNAs were extracted to quantify PB1 mRNA level (B) and PB2 mRNA level (C) by real-time RT-PCR. (D) The expression of PB2 was analyzed by Western Blot. Error bars standard error of the mean of four independent experiments.

We next investigated whether the expression of model vRNA in A549-5Ps cells is dependent on the presence of influenza RdRp and NP and, more importantly, whether the replication and expression of the viral minigenome mimic the same process that occurred during IAV infection. To demonstrate this, we first investigated the effect of disrupting IAV RdRp, e.g., knockdown PB1 expression, upon the expression of Gluc in A549-5Ps cells. A549-5Ps cells were transfected with PB1 siRNA and PB2 siRNA, respectively. A scramble siRNA (siNC) and irrelevant siRNA targeting influenza M2 were used as a negative control. Then the Gluc activity in the supernatant was measured (Fig 3A), and the amounts of PB1 mRNA (Fig 3B) and PB2 mRNA (Fig 3C) in the cells was quantified. As shown in Fig 3, the expression of Gluc was significantly decreased when the cells were transfected with PB1 siRNA and PB2 siRNA (Fig 3A), concomitant with reduced content of PB1 mRNA and PB2 mRNA, whereas treatment with either negative control siRNA or irrelevant M2 siRNA had no effect on either Gluc activity or PB1 mRNA level. The expression of PB2 was significantly decreased accompanied by reduced content of PB2 mRNA (Fig 3D). Therefore, these results demonstrate that Gluc expression in A549-5Ps cells specifically depends on the activity of influenza RdRp.

**Fig 4:**
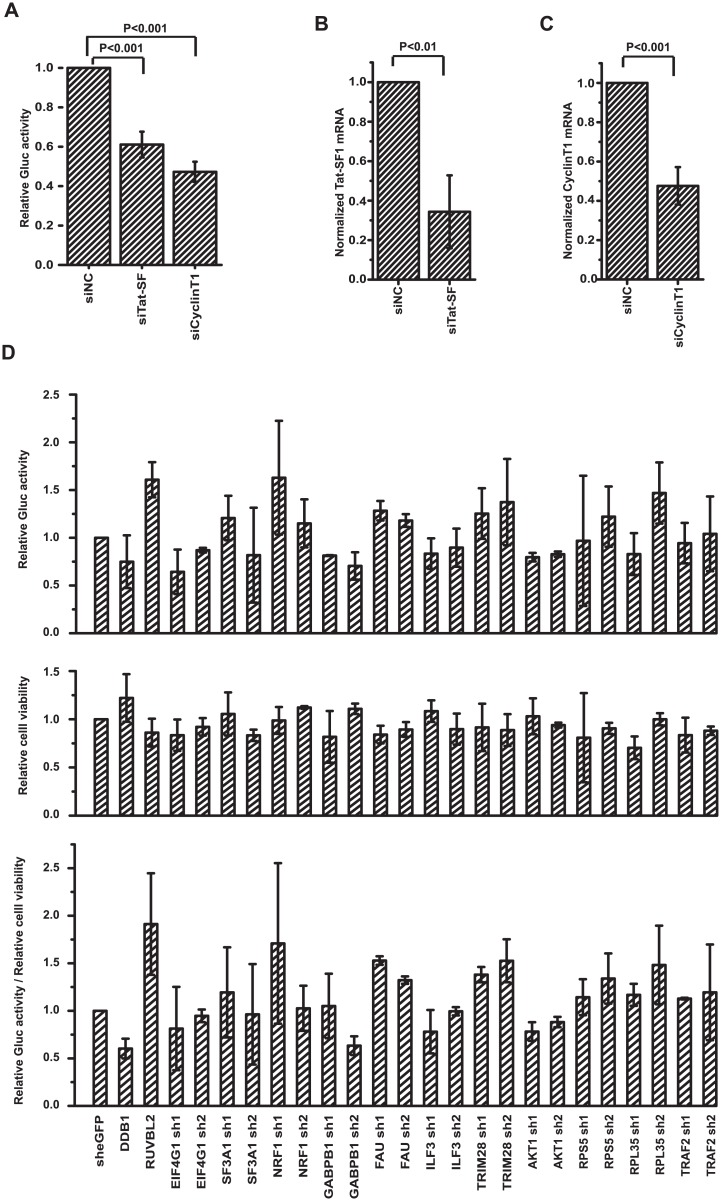
Effect of host factors knockdown on Gluc expression in A549-5Ps cells. A549-5Ps cells were transfected with 10nM negative control siRNA (NC), Tat-SF1 siRNA and cyclinT1 siRNA, respectively. (A) Gluc activity in supernatants was quantified. (B and C) Total cellular RNAs were extracted to quantify mRNA level of Tat-SF1 and cyclinT1 by real-time RT-PCR. Error bars standard error of the mean of four independent experiments. (D) A549-5Ps cells were transducted with shRNAs targeting candidate genes as indicated. The Gluc activity (top panel) and cell viability (middle panel) were quantified, and GLuc activity normalized with cell viability was presented in the bottom panel.

In addition to influenza RdRp, a large number of host factors involve in IAV RNA transcription/replication processes [8, 11, 29–35]. For example, it had been reported that Tat-SF1 stimulates the formation of influenza RNP complex [30] and cyclinT1/CDK9 facilitates the interaction between influenza RdRp and cellular RNA polymerase II [31]. Therefore, these host factors should be required for the expression of Gluc in the A549-5Ps cells, if the replication and expression of the viral minigenome mimic the same process that occurred during IAV infection. To verify this hypothesis, we investigated the effect of down-regulation of Tat-SF1 and cyclinT1 on the Gluc expression in A549-5Ps cells. A549-5Ps cells were transfected with siRNA-targeting Tat-SF1, cyclinT1 and negative control siRNA, respectively, followed by measuring Gluc activity and quantifying Tat-SF1 and cyclinT1 mRNA. The result showed a 40% and 53% decrease of Gluc activity in Tat-SF1 and cyclinT1 silencing A549-5Ps cells (Fig 4A), respectively, and a similar reduction in mRNA content was observed (Fig 4B and 4C), compared with cells treated with negative control siRNA. These results indicate that these cellular factors involved in the expression of Gluc in A549-5Ps cells, providing evidence supporting that the replication and expression of the viral minigenome mimic the same process that occurred during IAV infection.

We next explored the potential use of the A549-5Ps cell line on screening host factors targeting IAV genome transcription and replication stages. According to the genome-wide RNAi screening results [9–11, 35–37], 11 genes shown to be likely to involve in IAV replication were selected as candidates for further study using the minigenome system (listed in Fig 4D). A549-5Ps cells were transducted with shRNAs targeting candidate genes or negative control shRNA expressing siRNA specific for eGFP, respectively. It has been reported that RuvB-like 2 (RUVBL2) inhibits the replication of the IAV genome [38] whereas DNA damage-binding protein 1 (DDB1) is required for IAV polymerase activity [11]. Therefore, shRNAs that target RUVBL2 and DDB1 respectively, were used to confirm the reliability of the assay. Supernatants were replaced with fresh medium at 72 hours post transduction to eliminate the background. Cells were cultured for an additional 24 hours to analyze Gluc activity in the supernatant (Fig 4D, top panel). Meanwhile, cell viability was measured by a CCK-8 method (Fig 4D, middle panel). As shown in the GLuc activity normalized with cell viability (Fig 4D, bottom panel), RUVBL2 knockdown in A549-5Ps cells increased Gluc activity up to 1.91-fold compared with cells transducted with scramble shRNA, whereas DDB1 knockdown reduced Gluc activity by approximate 1.7-fold, which were consistent with previous reports [11, 38]. Among these candidates, the knockdown of SF3A1, FAU and TRIM28, respectively, increased Gluc activity, which suggests their inhibitory effect on the transcription and replication processes of IAV genome (Fig 4D). This work illustrates the application of the minigenome system in the elucidation of the role of host factors in IAV replication.

### Validation of the assay for HTS of inhibitors targeting IAV genome transcription and replication stages

**Fig 5:**
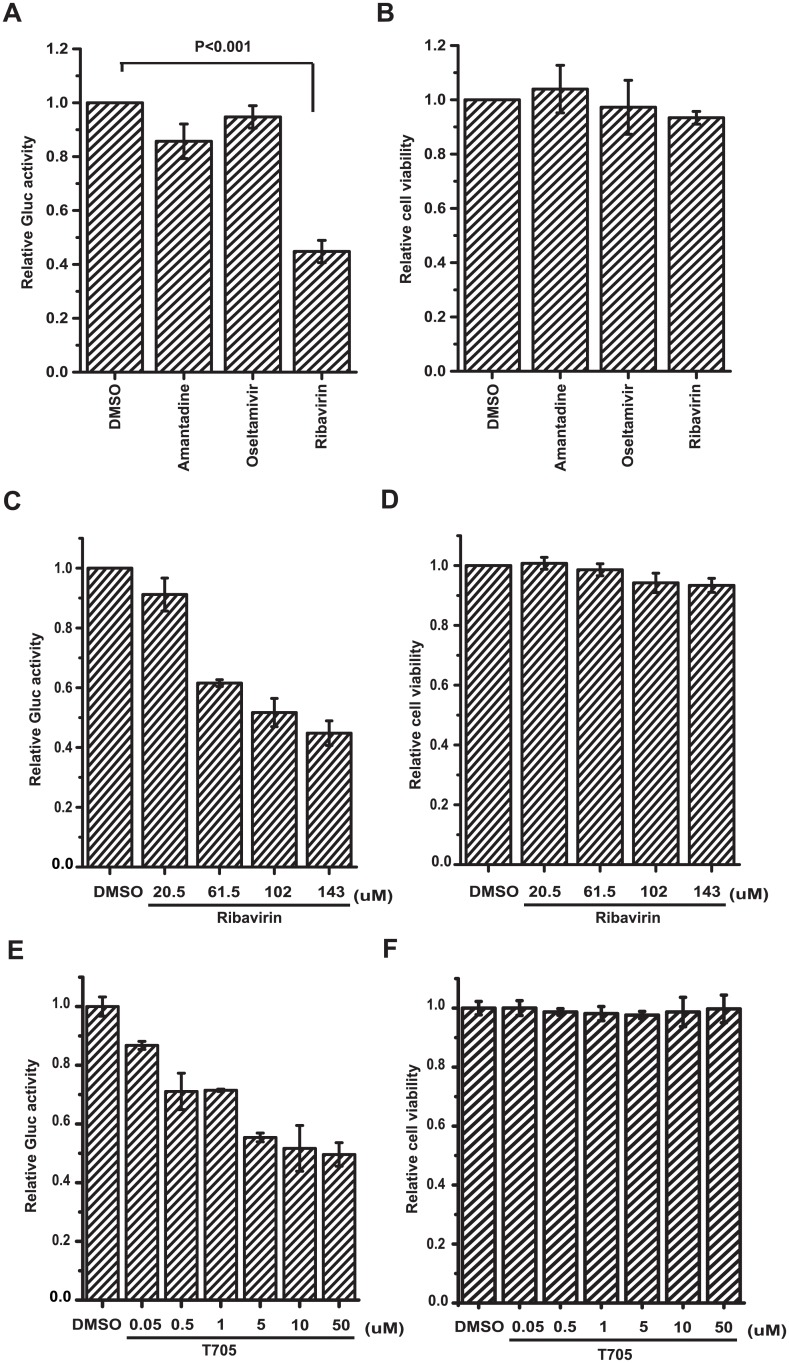
Inhibitory effects of known anti-influenza compounds on IAV minigenome transcription/replication system in A549-5Ps cells. (A-B) Inhibitory effects of amantadine hydrochloride, oseltamivir phosphate and ribavirin. A549-5Ps cells were treated with indicated compounds (50μM amantadine hydrochloride, 300μM oseltamivir phosphate or 143μM ribavirin) for 72 hours. (A) Gluc activity in supernatants was quantified. (B) Cell viability was analyzed using CCK-8 reagent. (C-D) Effects of ribavirin on IAV minigenome transcription/replication system. A549-5Ps cells were treated with various concentration of ribavirin for 72 hours, followed by Gluc activity analysis (C) and cell viability measurement (D). (E-F) Effects of T705 on IAV minigenome transcription/replication system. A549-5Ps cells were treated with various concentration of T705 for 72 hours, followed by Gluc activity analysis (E) and cell viability measurement (F). Error bars standard error of the mean of three independent experiments.

We next explored the potential use of the A549-5Ps cell line for screening inhibitors targeting IAV genome transcription and replication stages, using three known anti-influenza compounds including amantadine, oseltamivir and ribavirin. Amantadine functions as an inhibitor of IAV M2 ion channel [39]. Oseltamivir impairs the activity of IAV neuraminidase [40]. Ribavirin inhibits the replication and transcription of IAV RNA [41]. A549-5Ps cells were incubated with amantadine hydrochloride (50μM), oseltamivir phosphate (300μM) and ribavirin (143μM), respectively, each of which at the given concentration is able to inhibit the replication of IAV efficiently (data not shown). The result of Gluc activity assay showed that ribavirin inhibited transcription and replication of the viral minigenome efficiently, whereas amantadine hydrochloride and oseltamivir phosphate failed to show any inhibitory effects in A549-5Ps cells (Fig 5A). Even when the concentration of amantadine hydrochloride or oseltamivir phosphate was increased to a maximum cell-tolerable level, no inhibitory effects were observed (data not shown). Meanwhile, these compounds did not cause detectable cytotoxicity at the given concentration (Fig 5B). The fact that among three anti-IAV compounds, only IAV RdRp inhibitor ribavirin exhibited the inhibitory effect on the Gluc activity provides proof-of-concept evidence for the use of A549-5Ps in screening IAV RdRp inhibitors. Furthermore, the dose-response assessment was performed using ribavirin. The results showed that Gluc activity was inhibited by ribavirin in a dose-dependent manner (Fig 5C), while the cell viability was not affected in the given concentration range (Fig 5D). An IC_50_ value of 102μM was calculated, which agreed with previously published range of IC_50_ values. To further validate the application of A549-5Ps to screen RdRp inhibitors, we used T705, a specific RdRp inhibitor, and investigated its effect upon the GLuc activity. The result showed the T705 efficiently inhibited the GLuc activity in A549-5Ps (Fig 5E), which is closed to the data previously published [42]. Meanwhile, no cytotoxicity was observed in the concentration range used in the experiment (Fig 5F).

**Fig 6:**
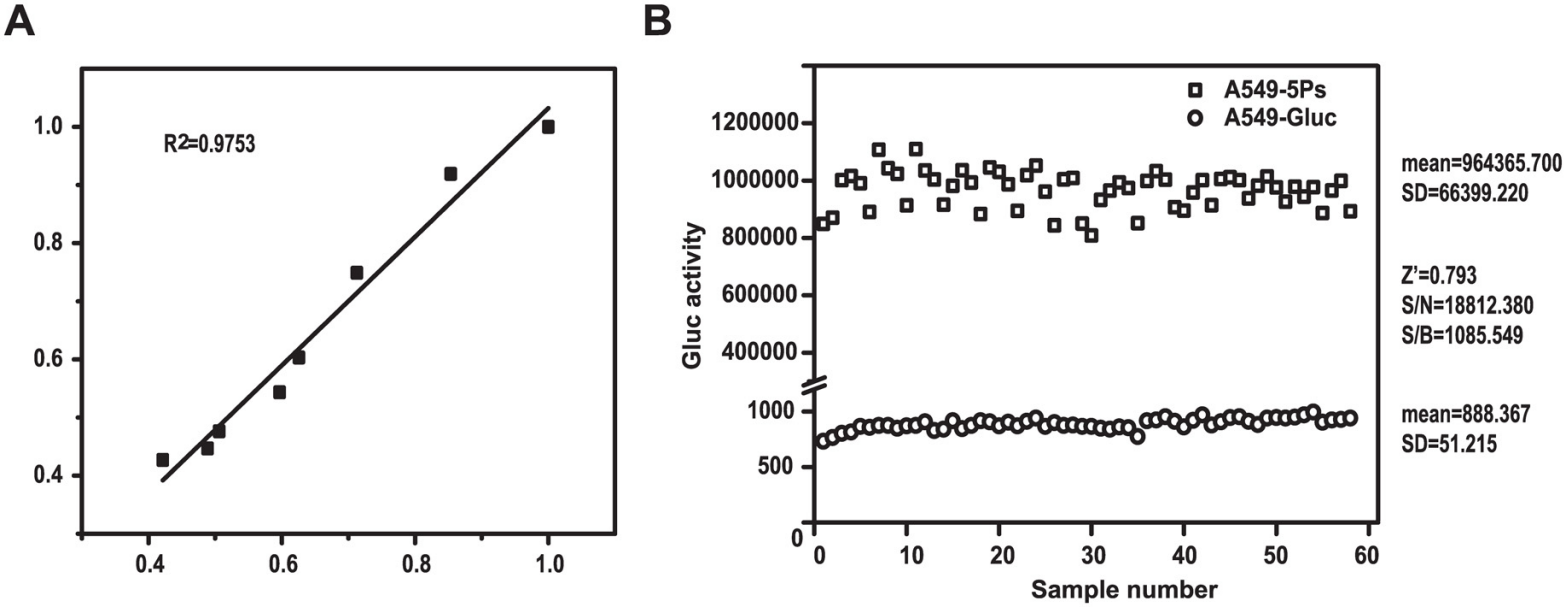
Validation of a HTS assay for IAV transcription/replication inhibitors. (A) Determination of R^2^ value. A549-5Ps cells were treated with various concentration of ribavirin for 72 hours, followed by Gluc activity analysis. R^2^ was calculated in two independent experiments (each experiment was triplicates). (B) Determination of parameters for the screening assay. A549-5Ps and A549-Gluc cells were seeded into 96-well plates (1×10^4^/well) and cultured for 72h hours. Supernatants were harvested to measure Gluc activity. Z’ factor, signal-to-noise ratio (S/N) and signal-to-background ratio (S/B) were calculated using the following equations: Z’ = 1−3×(SD of A549-5Ps + SD of A549-Gluc)/(mean of A549-5Ps − mean of A549-Gluc); S/N = (mean of A549-5Ps − mean of A549-Gluc)/SD of A549-Gluc; S/B = mean of A549-5Ps/mean of A549-Gluc.

To investigate the robustness of the assay, we conducted a study on the correlation of reproducibility of the assay using various amounts of ribavirin. Results obtained from two duplicated experiments were plotted against each other. The high R^2^ value (r^2^ = 0.97, P = 0.0001) indicates the high reproducibility of the assay (Fig 6A). Next, we utilized the Z’ factor to evaluate the quality of HTS assays. The Z’ factor is reported to reflect both the assay signal dynamic range and the data variation associated with the signal measurements [43]. To ensure the quality of the assay, it is recommended that other traditional assay performance measures, e.g. signal-to-noise ratio (S/N) and signal-to-background (S/B) ratio, should be used along with the Z-factor [44]. A549-Gluc and A549-5Ps cells were seeded into 96-well plates (1×10^4^ cells/well) and cultured for 72 hours, following by Gluc activity assay in supernatants. A549-GLuc is an A549 cell line that only contains the anti-sense Gluc/IRES/Bsd expression cassette without other components of RdRp. The results showed that the Z’ factor was 0.793, and S/N and S/B were much greater than the criteria required (Fig 6B), all of which meet the criteria for HTS assays. Taken together, these results demonstrate that this assay is suitable for HTS for inhibitors of IAV genome transcription and replication stages.

## Discussion

This work provides an ideal HTS assay to analyze the transcription/replication of IAV RNA, using A549-5Ps cells stably expressing IAV RdRp complex, NP and a viral mini-genomic RNA. The experimental evidence demonstrated that the expression of Gluc reporter was driven by influenza RdRp in A549-5Ps cells, and the replication and expression of the viral minigenome mimic the same process that occurred during IAV infection (Figs 3 and 4).

IAV genome transcription and replication are complicated biological processes involving the IAV RdRp complex, various known and unknown cellular components. In addition, these processes are characterized by several distinct features, such as RdRp activity and cap-snatching. Accordingly, many drug targets shall be expected to exist in the replication and expression of IAV genome RNA. However, there is no approved drug targeting influenza vRNA transcription/replication thus far, except for ribavirin, a broad-spectrum antiviral agent. Therefore, an ideal primary screening assay shall cover all potential targets to increase the number of hits. Indeed, our cell-based assay presented herein mimics all of the biochemical steps of viral genome transcription/replication, and the level of Gluc expression reflects the outcome of the entire process (Fig 1). The screening assay provides opportunities to identify hits with different mechanisms and to maximize the likelihood of finding novel anti-influenza drugs targeting viral genome transcription and replication stages.

To date, most published works use transient reconstitution of an IAV minigenome transcription/replication system to screen inhibitors. Recently, Ozawa et al reported a 293 cell line stably expressing influenza vRNPs, however a drug resistance gene used to monitor the activity of IAV RdRp causes low detection sensitivity [26]. In our assay, a secreted Gluc is utilized as the reporter gene, which has several advantages including high sensitivity, a wide linear detection range and convenience. Moreover, this test can be conducted no longer than 24 hours (Fig 2), resulting in impressive efficiency of the HTS assay. The statistical analysis presented herein further demonstrates the high reproducibility of the assay (Fig 6).

In summary, we established and validated a simple, convenient, and reliable HTS system to monitor IAV minigenome transcription and replication, which will promote the development of novel anti-influenza drugs and identification of new host factors involved in influenza RNA transcription and replication.
